# Asian tiger mosquito in the oil-producing city of Soyo: the first report of *Aedes* (*Stegomyia*) *albopictus* (Skuse, 1894) in Angola

**DOI:** 10.1186/s13071-025-06741-y

**Published:** 2025-03-05

**Authors:** José Franco Martins, Arlete Dina Troco, Cátia Marques, Vicente Chipepa, Gonçalo Seixas, João Pinto, Luzala Garcia, Cani Pedro Jorge, Eusébio Manuel, Gonçalo Alves

**Affiliations:** 1 National Malaria Control Program, National Directorate of Public Health, Ministry of Health, Luanda, Angola; 2 The Mentor Initiative, Luanda, Angola; 3 Population Services International, Luanda, Angola; 4 Global Health and Tropical Medicine, GHTM, Associate Laboratory in Translation and Innovation Towards Global Health, LA-REAL, Instituto de Higiene e Medicina Tropical, UNL, Lisbon, Portugal; 5 Department of Hygiene and Epidemiological Surveillance, National Directorate of Public Health, Ministry of Health, Luanda, Angola; 6 The Mentor Initiative, Haywards Heath, UK

**Keywords:** *Aedes albopictus*, Invasive mosquito, Mosquito surveillance, Angola

## Abstract

**Background:**

The Asian tiger mosquito, *Aedes albopictus* (Skuse, 1894), is a highly invasive species that has successfully colonized many tropical and temperate regions worldwide. Its rapid global spread is strongly associated with human activities and has created favorable conditions for the emergence of human arboviruses in new geographic areas.

**Methods:**

Mosquito larvae were collected by community health workers from different breeding sites and reared to adults in a field insectary. Adult mosquitoes were morphologically identified to species level. Species identification was confirmed by cytochrome oxidase subunit I DNA barcoding.

**Results:**

We report the first detection of *Aedes albopictus* in Angola during an *Anopheles stephensi* survey conducted in Soyo, Zaire Province. Phylogenetic analysis indicated that the Angolan *Ae. albopictus* population clusters with sequences from Central African countries, suggesting an introduction from within the continent.

**Conclusions:**

The presence of *Ae. albopictus* in Angola highlights the need for enhanced vector surveillance and control measures to prevent the emergence of arboviral diseases. This finding emphasizes the relevance of collaboration between local health authorities, communities, and international organizations in monitoring the spread of invasive mosquito species.

## Background

*Aedes albopictus* (Skuse, 1894) is a mosquito native to Southeast Asia and is one of the most invasive species, having successfully colonized many tropical and temperate regions [1]. Genetic evidence suggests that this rapid global spread is strongly associated with human activities, particularly the international trade of used tires through maritime and land routes [2, 3]. The ecological adaptability of *Aedes* species enables their proliferation across diverse climates and habitats [4]. Notably, *Aedes* species, such as *Aedes aegypti* and *Aedes albopictus,* serve as primary vectors of arboviral diseases such as dengue [5, 6]. The presence of *Ae. albopictus* has created conditions conducive to the emergence and spread of human arboviruses, such as chikungunya, Zika, dengue, and yellow fever in new geographic areas [7]. Epidemiological surveillance has confirmed the circulation of dengue [8–11], chikungunya [8, 12], and Zika virus [13] in Angola, in Luanda (capital), as well as in other provinces. These arboviruses have been primarily associated with the presence of *Ae. aegypti* mosquitoes. The coexistence of *Ae. aegypti* and *Ae. albopictus* has been associated with an increased risk of arbovirus transmission, as both species are competent vectors for dengue, chikungunya, and Zika viruses. Studies have shown that in regions where both species are present, there is an extended seasonal transmission period and higher virus circulation owing to their differing ecological niches and biting behaviors. For instance, *Ae. albopictus* has demonstrated the ability to sustain transmission in peri-urban and rural areas, complementing the role of *Ae. aegypti* in urban environments [7, 14]. This dual-vector scenario has led to more frequent and intense outbreaks in regions where both mosquitoes have established populations [3]. Understanding this dynamic is crucial in the context of Angola, where the introduction of *Ae. albopictus* may contribute to an increased arboviral burden. Over the last 30 years, these arboviruses have expanded significantly in both distribution and public health impact [15]. The spread of *Ae. albopictus* is associated with considerable environmental and economic costs, although the full extent of these impacts remains to be comprehensively assessed [16].

In continental Africa, *Ae. albopictus* was first recorded in South Africa in 1989 [17]. In Central Africa, *Ae. albopictus* was reported in Cameroon in 2000 [18] and has since spread throughout the region, including into two countries bordering Angola, the Democratic Republic of the Congo [19] and Zambia [20]. This expansion in Central Africa coincided with outbreaks of dengue, Zika, and chikungunya in urban areas, highlighting the potential epidemiological consequences of its establishment in Angola [21]. Angola was one of the few African countries where *Ae. albopictus* had not yet been detected [22].

The National Malaria Control Program (NMCP), in partnership with international nongovernmental organizations such as Population Services International (PSI) and The Mentor Initiative (MI), has been leading efforts to enhance malaria vector surveillance in Angola. As part of these initiatives, a community-based mosquito surveillance approach was established in Zaire Province under the Health For All (HFA) project. This initiative focused on active surveillance of the invasive malaria vector *Anopheles stephensi* (Liston, 1901), targeting ports and neighboring communities to enhance early detection. Community health workers (CHW) received training in standardizing mosquito collection techniques for both adult and immature specimens. Upon successful completion of their training, CHWs were equipped with necessary tools and consumables to facilitate regular mosquito collections. These efforts are aligned with World Health Organization (WHO) policies on effective vector-borne disease control through sustainable and locally adapted interventions [23].

Here, we report for the first time the detection of *Ae. albopictus,* identified through entomological surveillance activities originally designed for *An. stephensi* monitoring in Soyo, Zaire Province.

## Methods

### Sampling area and mosquito collections

The northwestern Angolan Province of Zaire is bordered by the Atlantic Ocean to the west, the River Congo and Democratic Republic of Congo to the north, Uíge Province to the east, and Bengo Province to the south. Soyo, located in Zaire Province, has emerged as a significant oil-producing city in Angola and is also home to the prominent Kwanda seaport. The local climate is predominantly tropical with both wet and semi-arid conditions. From February to April 2024, as part of the HFA entomological surveillance targeting *An. stephensi*, mosquito breeding sites in Soyo were surveyed, with a particular focus on ports and surrounding communities. The collections were carried out by trained CHWs with formative supervision from the NMCP, MI, and PSI. Larval collections were done using 350 ml standard mosquito dippers (BioQuip Products, Rancho Dominguez, CA, USA) and plastic pipettes. The survey included natural and artificial breeding sites. Natural breeding sites consisted of rain puddles, while artificial breeding sites included containers, such as plastic bottles, discarded tires, and metal drums. None of the breeding sites surveyed were used for water storage. Collected larvae were brought to the field insectary and reared to the adult stage. The larvae were fed with commercial tropical fish flakes. After emergence, adult mosquitoes were kept in an entomological cage until morphological identification. The adult mosquitoes were maintained on a 10% sugar solution.

### Morphological identification

Following emergence, adult mosquitoes were morphologically identified using standardized mosquito identification keys [24–26]. After identification, mosquitoes were placed individually or in pools of five to seven specimens in labeled 1.5 ml microtubes containing silica gel and stored at room temperature for molecular analysis.

### *Aedes albopictus* molecular analysis

Genomic DNA of both female and male *Ae. albopictus* were extracted from whole mosquitoes according to Collins et al. [27]. Extracted DNA was subjected to cytochrome oxidase subunit I (COI) barcoding using the primers LCO1490 (5′-GGTCAACAAATCATAAAGATATTGG-3′) and HCO2198 (5′-TAAACTTCAGGGTGACCAAAAAATCA-3′) [28]. The PCR reaction mixture contained 1 µl of the extracted DNA, 1 µl of each primer at 0.5 mM, and 10 µl of NZYTaq II 2× Green Master Mix (NZYtech), to a final volume of 20 µl obtained with double-distilled (dd)H_2_O. All PCR assays included negative controls (no DNA template). The PCR thermal conditions were as follows: initial denaturation at 94 °C for 4 min, followed by 30 cycles of denaturation at 94 °C for 40 s, annealing at 50 °C for 60 s, extension at 72 °C for 60 s, and a final extension step at 72 °C for 5 min. Five microliter aliquots of the obtained PCR products were size fractionated by electrophoresis on 1% agarose gels stained with GreenSafe Premium (NZYtech). The remaining amplified products were purified and submitted to Sanger sequencing at STABVIDA (Caparica, Portugal). For independent confirmation and validation, nine *Ae. albopictus* specimens were sent to the Centers for Disease Control and Prevention (CDC) in Atlanta for COI DNA barcoding [28]. The resulting sequences were edited and aligned using BioEdit (version 7.7.1) [29]. Similarity searches were performed in GenBank (NCBI, www.ncbi.nlm.nih.gov) using the BLASTN algorithm [30]. A phylogenetic analysis was conducted using the neighbor-joining method in MEGA X [31] to determine relatedness of our sequences (accession numbers in Results section) to available sequences at GenBank database [30]. To construct the phylogenetic tree, 35 sequences of *Ae. albopictus* were downloaded, and the metadata of each sequence were sorted on the basis of country.

## Results

### *Aedes albopictus* occurrence

**Fig. 1:**
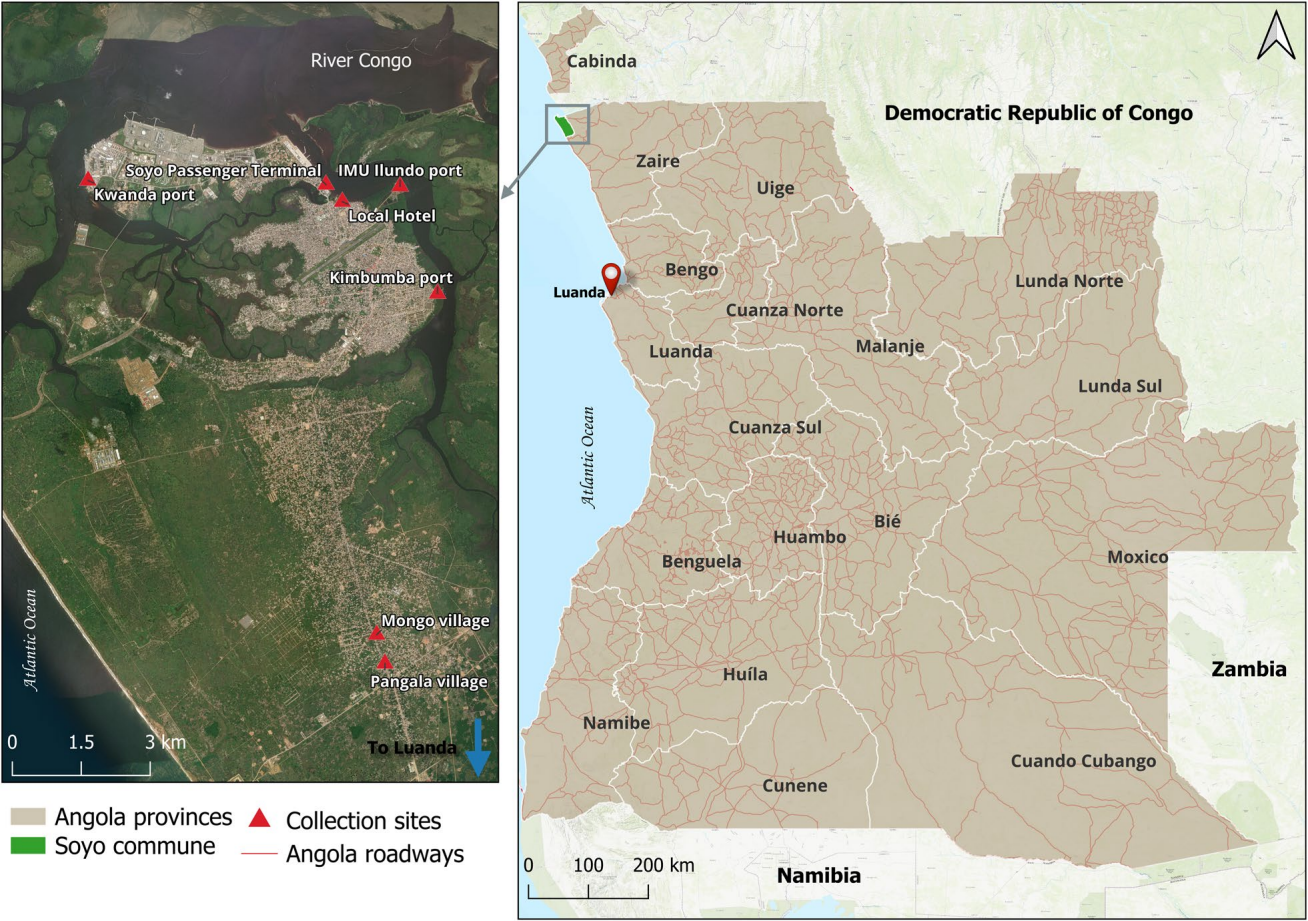
Immature mosquito collection sites in Soyo commune, Zaire Province, Angola. Map generated using QGIS (version 3.38.3); Open-Source Geospatial Foundation Project

From February up to the end of April 2024, breeding sites were surveyed in seven collection sites across the commune of Soyo (Fig. 1). All sites were located along the road crossing the city and connecting Soyo to the rest of the country, including Luanda. On 21 February 2024, CHWs brought from the field a mixed collection of *Aedes* and *Anopheles* larvae and unidentified pupae collected in plastic bottles and metal drums used as a flower nursery in a local Hotel in the city Soyo. On February 22, two females and one male emerged from the collected pupae. The three specimens were morphologically identified as *Ae. albopictus*. A total of 674 mosquito larvae and pupae were collected across the seven collection sites until the end of April. After adult emergence, 252 mosquitoes were morphologically identified as *Ae.* *albopictus*, 100 as *Aedes aegypti* (Linnaeus, 1762) and 143 as *Anopheles gambiae* s.l. (Giles, 1902) (Table 1). The remaining 179 adult mosquitoes were identified only up to genus level (92 *Anopheles* spp. and 87 *Aedes* spp.).

**Table 1:** Morphological identification of adult mosquitoes collected from Soyo

Collection sites	Morphological ID															Total
	*Ae. albopictus*			*Ae. aegypti*			*An. gambiae* s.l			*An. spp*.*			Ae. spp.*			
	♀	♂	Sum	♀	♂	Sum	♀	♂	Sum	♀	♂	Sum	♀	♂	Sum	
Kwanda seaport	5	2	7	3	–	3	–	–	0	4	–	4	24	12	36	50
Soyo passenger terminal	–	1	1	4	2	6	7	6	13	–	–	0	17	6	23	43
Local hotel	51	24	75	29	22	51	10	–	10	–	–	0	–	–	0	136
IMU Ilundo port	129	18	147	0	2	2	22	6	28	51	37	88	20	8	28	293
Kimbumba port	–	–	0	–	–	0	57	35	92	–	–	0	–	–	0	92
Mongo village	3	2	5	25	5	30	–	–	0	–	–	0	–	–	0	35
Pangala village	10	7	17	7	1	8	–	–	0	–	–	0	–	–	0	25
Total	198	54	252	68	32	100	96	47	143	55	37	92	61	26	87	674

*Samples of adult mosquitoes only identified to genus level

### Molecular identification and phylogenetics analysis

**Fig. 2:**
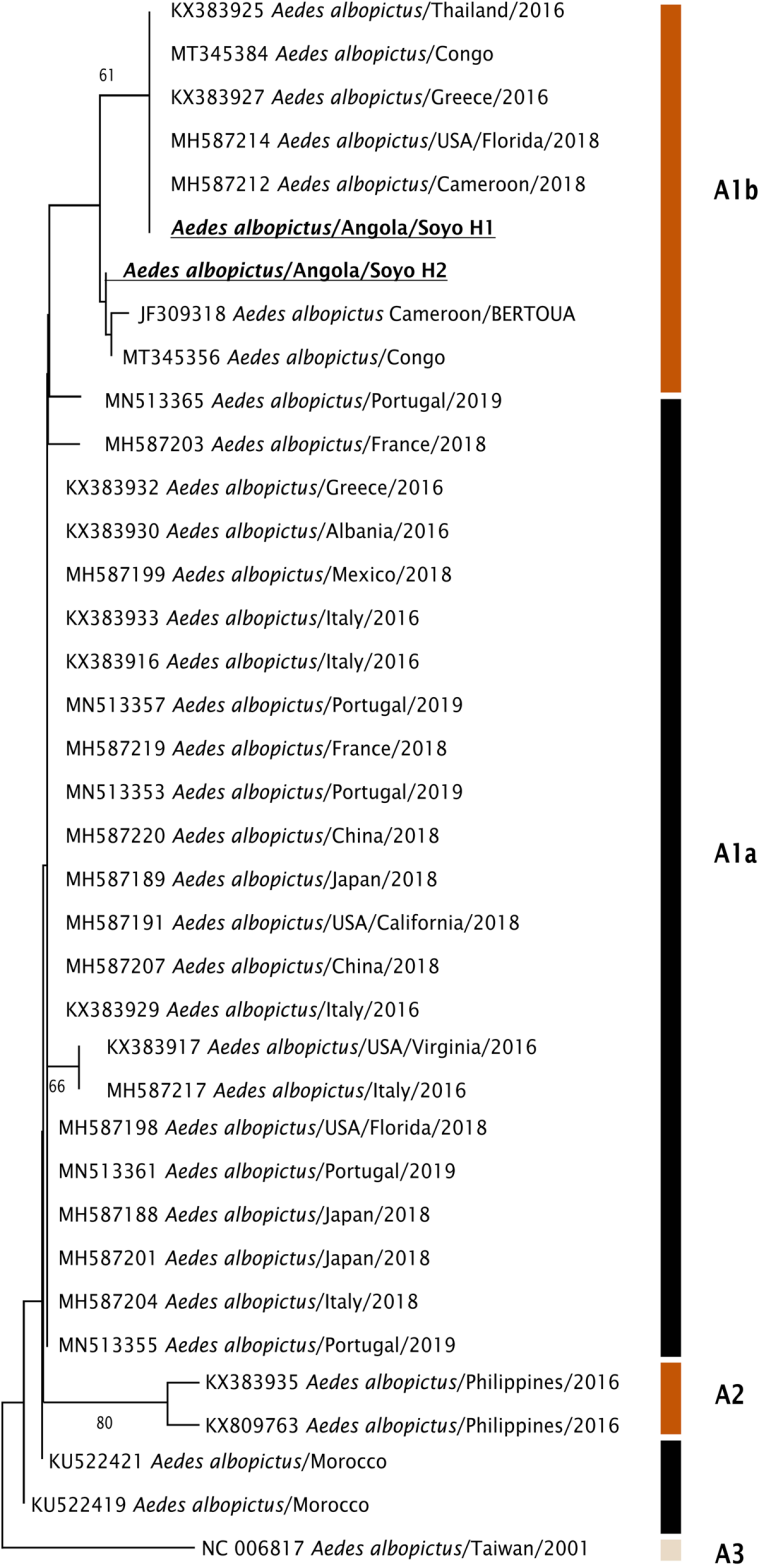
Phylogenetic analysis based on partial mitochondrial cytochrome c oxidase subunit I (COI) sequences of *Ae. albopictus*. The analysis was conducted in MEGA X using the neighbor-joining method. The evolutionary distances were computed using the Kimura two-parameter method in the bootstrap test (1000 replicates). Bootstrap numbers under 60 were omitted. A total of 649 bp were applied

We conducted COI DNA barcoding analysis on 38 specimens previously identified morphologically as *Ae. albopictus*. Molecular identification confirmed all specimens as *Ae. albopictus* (99.0–100.0% identity). Sequencing revealed two distinct haplotypes, H1 (*Aedes albopictus*/Angola/Soyo H1; accession no. PQ156979) and H2 (*Aedes albopictus*/Angola/Soyo H2; accession no. PQ156980), indicating low genetic variability. Haplotype H1 was more prevalent, constituting 92.1% (*n* = 35) of the total mosquitoes sequenced. Haplotype H2, representing only 7.90% (*n* = 3) of the sequenced samples, consisted of two females collected in the local hotel and one male collected from Pangala village (Table 2). To further investigate the genetic relationships and putative geographical origins, we constructed a phylogenetic tree using the COI sequences of the identified haplotypes, H1 and H2 (Fig. 2). Our analysis indicated that the two haplotypes clustered with sequences of clade A1b [32].

**Table 2:** Haplotypes of *Aedes albopictus* from Soyo by sex and sampling site

Haplotypes H, accession no.	Sex	Collection sites				Total, *n* (%)
		Local hotel	Mongo village	Pangala village	IMU Ilundo port	
H1, PQ156979	♀	9	2	6	8	35 (92.1)
	♂	5	2	2	1	
H2, PQ156980	♀	2	–	–	–	3 (7.90)
	♂	–	–	1	–	
Total		16	4	9	9	38 (100.0)

## Discussion and conclusions

In this work we confirm, through morphological and molecular identification methods, the presence of *Ae. albopictus* in the commune of Soyo in the northern Province of Zaire in Angola. Given the species’ ongoing expansion across the continent and its presence in neighboring Democratic Republic of Congo [33], its detection in Angola was anticipated.

The low genetic variability found is indicative of a founder effect [34]. While this suggests a recent introduction of *Ae. albopictus* into Angola, it does not allow us to determine the precise date of its arrival to the area of Soyo. The phylogenetic analysis shows that the Angolan *Ae. albopictus* clusters with sequences from Cameroon and the Democratic Republic of Congo, which align with the clade A1b [32]. These findings indicate a possible introduction route from neighboring countries. Interestingly, the Angolan haplotype H2 was previously described in São Tomé Island (haplotype ST3), located in the Gulf of Guinea, suggesting potential connectivity between these regions [35].

The presence of *Ae. albopictus* in Angola raises concerns regarding its role in local transmission of arboviruses, namely dengue, Zika, and chikungunya. While *Ae. aegypti* remains the primary vector of these viruses in Angola, the establishment of *Ae. albopictus* could alter transmission dynamics and increase the risk of outbreaks. The coexistence of these species complicates vector control efforts and underscores the need for comprehensive mosquito surveillance [3, 36]. For Angolan health authorities, the presence of *Ae. albopictus* is of significant public health relevance owing to its capacity to transmit arboviral diseases [5]. According to the 2022 WHO [37] report on national capacities to respond to arboviral diseases, Angola demonstrates a robust capacity to manage these diseases, achieving a score of 73.4% (scale of 0–100%). However, epidemiological data remain limited, preventing a comprehensive assessment of the current risk [11]. Dengue remained relatively unchanged from 2288 cases in 2020 to 2710 in 2023, while chikungunya cases decreased from 3794 in 2020 to 1500 in 2023. From 2020 to 2023, Zaire Province, where *Ae. albopictus* was first detected, reported 84 cases of dengue fever and 225 cases of chikungunya [38].

Further studies are needed to determine the distribution, ecological adaptability, insecticide susceptibility, and vector competence of the Angolan population of *Ae. albopictus*.

The detection of *Ae. albopictus* under a project focused on malaria entomological surveillance highlights the importance of an integrated mosquito management (IMM) approach. Monitoring efforts that focus solely on single disease vectors monitoring may overlook significant emerging threats posed by other mosquito species. A more ambitious approach would involve the expansion of vector surveillance to encompass multiple species under an integrated vector management (IVM) program. Such an initiative should engage multiple MoH health programs, nongovernmental organizations, academic institutions, and the private sector.

In this work, we demonstrate how collaborative partnerships, guided by the leadership of Angolan national health authorities, can enhance mosquito surveillance efforts. Our findings highlight the importance of regular entomological monitoring not only for established vectors but also for newly introduced species such as *Ae. albopictus*. Angola is already working toward early detection of *An. stephensi* at potential entry points, such as the seaports in Soyo (Zaire province), Luanda (Luanda province), and Lobito (Benguela province).

Implementing an IVM strategy will be crucial for the early detection of invasive mosquito species and the timely implementation of effective control measures.

## Data Availability

The data and materials that support the fi ndings of this study are available from the corresponding authorupon request. Sequences have been submitted to NCBI Genbank database.

## Abbreviations

CHW: Community health workers
BLASTN: Basic local alignment search tool nucleotide
COI: Cytochrome oxidase subunit I
DNSP: National Directorate of Public Health
IMM: Integrated mosquito management
IVM: Integrated vector management
MI: The Mentor Initiative
MoH: Ministry of Health
NCBI: National Center for Biotechnology Information
NMCP: National Malaria Control Program
PCR: Polymerase chain reaction
PSI: Population services international
WHO: World Health Organization
